# An Incidentally Discovered Large Left Main Coronary Artery Aneurysm

**DOI:** 10.7759/cureus.9172

**Published:** 2020-07-13

**Authors:** Mohammed Elsadany, Jared Selter, Joseph Mattana

**Affiliations:** 1 Internal Medicine, St. Vincent's Medical Center, Bridgeport, USA; 2 Internal Medicine, Frank H. Netter MD School of Medicine, North Haven, USA; 3 Cardiology, St. Vincent's Medical Center, Bridgeport, USA

**Keywords:** left main coronary artery aneurysm, left main aneurysm

## Abstract

Left main coronary artery aneurysms (LMCAA) are rare. The most common cause is atherosclerosis followed by congenital malformations. Patients with LMCAA can present with shortness of breath and angina if there is coexisting obstructive coronary artery disease. Here we describe a patient incidentally found to have a 2 cm aneurysm of the left main coronary artery in the setting of an ST-elevation myocardial infarction (STEMI) and we discuss potential medical and surgical treatment options for this incompletely understood condition.

## Introduction

Coronary artery aneurysm (CAA) is defined as dilatation of a coronary artery segment to more than 1.5-fold its normal diameter. It is uncommon with an incidence rate from 0.3% to 5.3% [1]. Left main CAA (LMCAA) is rare with a reported incidence rate of 0.1% [2,3]. Patients with LMCAA can present with ischemic heart disease symptoms if there is coexistent obstructive coronary artery disease within the aneurysm, but others may present with symptoms unrelated to the aneurysm. We describe a patient with a large LMCAA which was detected incidentally during cardiac catheterization after the patient presented with an ST-elevation myocardial infarction (STEMI) secondary to complete occlusion of the mid-right coronary artery.

## Case presentation

**Figure 1:**
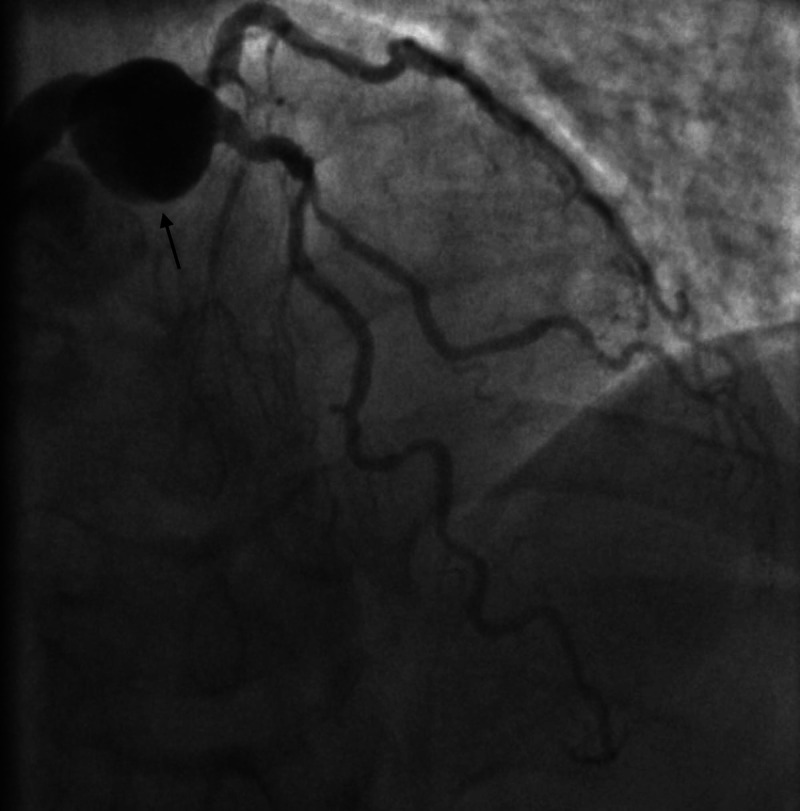
Left heart catheterization RAO cranial view showing left main coronary artery aneurysm and 75% stenosis in the first diagonal. RAO: right anterior oblique.

**Figure 2:**
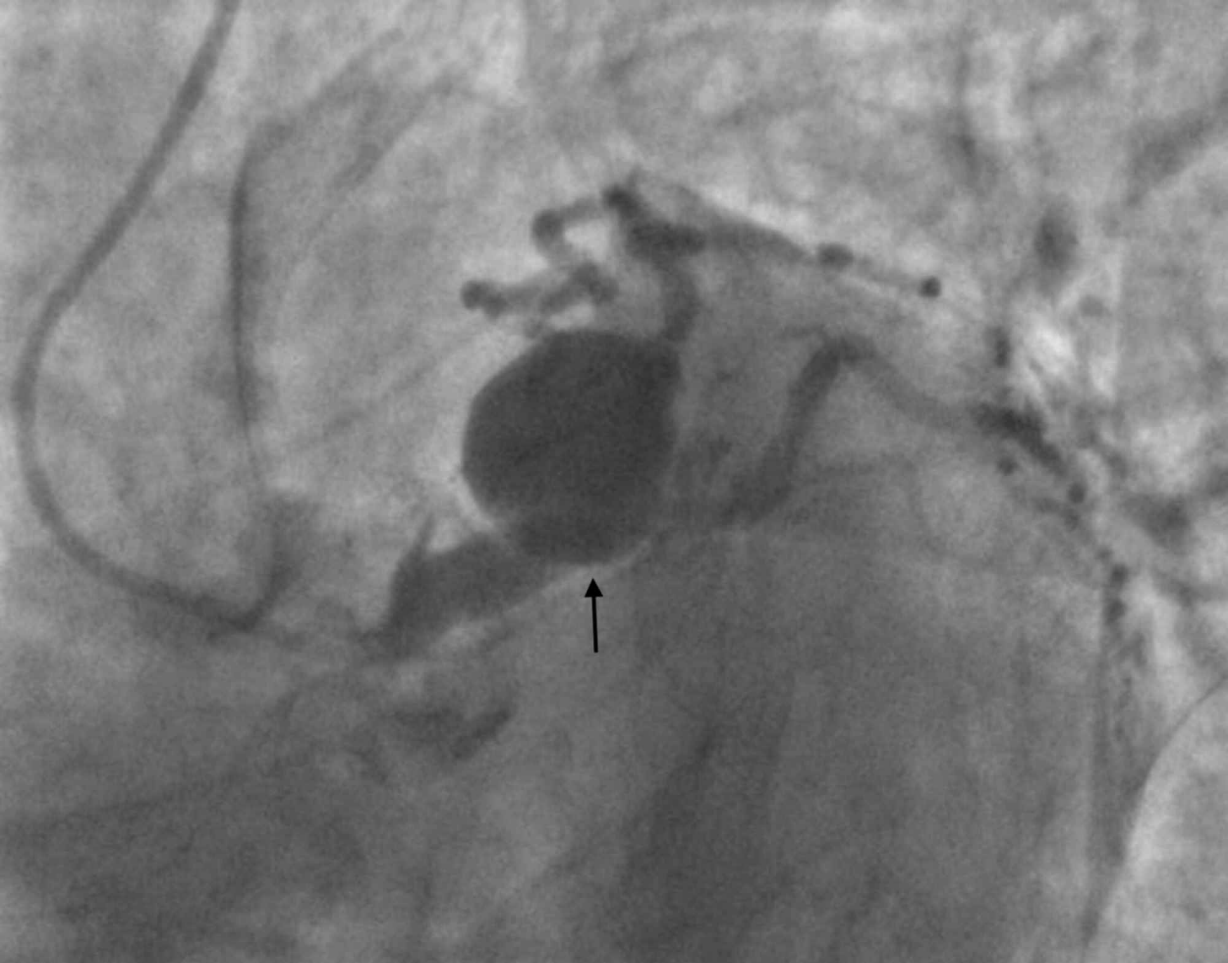
Left heart catheterization LAO caudal view showing left main coronary artery aneurysm. LAO: left anterior oblique

A 64-year-old woman with a history of hypertension presented to the emergency department with sudden onset mid-sternal chest discomfort. Electrocardiography confirmed an acute inferior wall STEMI and she underwent emergent cardiac catheterization. Angiography showed a 75% stenosis in the first diagonal and complete occlusion of the mid-right coronary artery which was the cause for the patient's inferior wall STEMI and was successfully revascularized with the placement of a single bare-metal stent. In addition, a 2 cm saccular aneurysm was noted in the distal left main (LM) coronary artery (Figures 1-2). The LM bifurcation was obscured by the aneurysm and a high-grade stenosis could not be excluded. Intravascular ultrasonography was not performed given the high risk of perforation. Surgical intervention was not pursued during her hospitalization given her recent infarct and equivocal LM findings. Her post-infarct course was uneventful, and she was discharged on a regimen of aspirin, ticagrelor, carvedilol, and atorvastatin.

## Discussion

CAA is defined as dilatation of a coronary artery segment to more than 1.5-fold its normal diameter. CAA is uncommon with an incidence rate from 0.3% to 5.3% [1]. Aneurysms can be either focal or diffuse and are classified according to morphology: fusiform or saccular. The most commonly affected coronary artery is the right (40%-87% of aneurysms). The involvement of the LM, as found in our patient, is rare with an incidence of only 0.1% [2,3]. The most common cause of CAA in adults is atherosclerosis (50% of cases) followed by congenital malformations (20%-30% of cases) [2]. Other causes include vasculitis, Marfan syndrome, traumatic injury, and idiopathic. Coronary angiography is the gold standard for diagnosis [4].

Patients with CAA are usually asymptomatic, though patients can present with shortness of breath and angina when obstructive coronary artery disease exists within the aneurysm. Furthermore, abnormal flow within a CAA may predispose to thrombus formation and distal embolization leading to myocardial infarction without the presence of obstructive coronary artery disease [5]. Our patient’s STEMI was unrelated to her CAA. 

The treatment of CAA is not well established due to the rarity of the condition. Treatment recommendations are based on case reports and expert consensus. Surgical intervention is indicated in patients with coexistent obstructive coronary artery disease within the aneurysm if myocardial infarction develops secondary to embolic events from the aneurysm, or in cases of progressive CAA enlargement documented by serial coronary imaging. Surgical interventions in LMCAA are usually performed by proximal and distal ligation of the aneurysm with bypass grafts to the left anterior descending and left circumflex coronary arteries. For medical treatment, current reports recommend starting antiplatelet and anticoagulation therapy to prevent thrombus formation within the aneurysm [6]. There is considerable uncertainty as to the optimal approach in a patient such as ours, given her need for dual antiplatelet therapy and increased risk of bleeding.

## Conclusions

CAA, especially LMCAA, is uncommon, with atherosclerosis being the most common etiology. Given its rarity, treatment of LMCAA is not well established, though surgical intervention is recommended if obstructive coronary artery disease is present within the aneurysm, if distal embolization with myocardial infarction occurs or if the aneurysm progressively enlarges. Recommended medical treatment includes antiplatelet and anticoagulation therapy to prevent thrombus formation within the aneurysm. Further study of this incompletely understood condition will hopefully lead to better guidance for optimal management of the scenario our patient presented with.
